# Development of topical ophthalmic *In Situ* gel-forming estradiol delivery system intended for the prevention of age-related cataracts

**DOI:** 10.1371/journal.pone.0172306

**Published:** 2017-02-21

**Authors:** Udaya K. Kotreka, Vicki L. Davis, Moji C. Adeyeye

**Affiliations:** Graduate School of Pharmaceutical Sciences, Duquesne University, Pittsburgh, PA, United States of America; Boston University School of Medicine, UNITED STATES

## Abstract

The goal of this study was to develop and characterize an ion-activated *in situ* gel-forming estradiol (E_2_) solution eye drops intended for the prevention of age-related cataracts. Accordingly, *in situ* gelling eye drops were made using gellan gum as an ion-activated gel-forming polymer, polysorbate-80 as drug solubilizing agent, mannitol as tonicity agent, and combination of potassium sorbate and edetate disodium dihydrate (EDTA) as preservatives. The formulations were tested for the following characteristics: pH, clarity, osmolality, antimicrobial efficacy, rheological behavior, and *in vitro* drug release. Stability of the formulation was also monitored for 6 months at multiple storage conditions per ICH *Q1A (R2)* guidelines. The solution eye drops resulted in an i*n-situ* phase change to gel-state when mixed with simulated tear fluid (STF). The gel structure formation was confirmed by viscoelastic measurements. Drug release from the gel followed non-fickian mechanism with 80% of drug released in 8 hr. The formulations were found to be clear, isotonic with suitable pH and viscoelastic behavior and stable at accelerated and long-term storage conditions for 6 months. *In vitro* results suggest that the developed formulation is suitable for further investigation in animal models to elucidate the ability of estrogen to prevent and delay cataracts.

## Introduction

A cataract is defined as a clouding of the eye's natural lens. In normal eyes, the lens focuses the incident light on the retina to activate photoreceptors and cause vision. In cataractous eyes, due to the clouding of lens, the incident light is scattered causing blurred vision [1]. Among the 285 million who are visually impaired globally, 33% (about 95 million) of the cases are due to cataract [2, 3]. It occurs more frequently in older population (> 50 years old). It is the leading cause of blindness in 116 countries [4]. In the US alone, the Center for Disease Control (CDC) estimated that 15 million Americans aged 65 years or older have a cataract in one or both eyes. By 2020, the estimated number of people aged 40 or older with cataracts is expected to rise to more than 30 million [5, 6]. The factors that lead to cataract formation include age, diabetes mellitus, cigarette smoking, elevated body mass index, UV light, and alcohol use [7, 8]. Since age is the single greatest factor, everyone is at risk of developing cataracts. More than 75% of people ≥75 years old have some degree of lens opacification, and it is estimated that >50% of blindness is caused by cataracts as mentioned above. There are currently no treatments that delay or prevent their occurrence, except surgical removal after a cataract progresses to the point it obscures vision significantly. The surgery is associated with risks, especially in elderly patients and often leads to the complication known as secondary cataracts. Moreover, in many countries there are barriers such as poverty, lack of access to medical care [2] and perceptions [9] that prevent patients to access surgery.

Epidemiological studies carried out to investigate the role of age and gender on the prevalence of cataracts indicated that the incidence of cataracts in postmenopausal women was higher relative to men of similar age [10–14], which may be due to low circulating levels of estrogen that occur after menopause. Also, supplementing postmenopausal women with estrogen through hormone therapy (HT) has resulted in reduced cataract risk, which further suggests a possible role for this class of female hormones in protecting against cataracts [8, 15–18]. 17β-estradiol (E_2_) is the most potent estrogen that occurs naturally in the human body. It has been reported to protect against loss of lens transparency in women [8] and in various rodent models [19–21]. However, many women are concerned about estrogen treatments due to the potential increased risk of breast and endometrial cancers that have been associated with elevated systemic levels of estrogen, which can occur via different drug delivery systems, such as through the oral [22, 23], buccal [24], vaginal [25], nasal [26], and transdermal [27, 28] routes.

The presence of estrogen receptors (ER) in rat, bovine, and human retina and rat, mouse, canine, and human lens suggests that eye can respond directly to estrogen [29–34]. Since the lens is an avascular tissue, delivery via systemic routes would be expected to be inefficient. Hence, a topical ocular delivery of estrogen that will allow localized interactions with its receptors in the lens to elicit protective responses would be a more efficient way to treat or prevent cataracts. In addition, ocular estrogen therapy would offer an attractive alternative to surgery, which, so far, is the only available treatment for cataracts

Drug delivery to ocular mucosa for local treatment is associated with great possibilities, but often also with many challenges. The physiological constraints imposed by the protective mechanisms of the eye also result in low absorption of drugs [35]. In addition, the most common means to deliver drugs to the eye is via instillation of an aqueous solution of the drug. However, the bioavailability of a drug introduced in this way is often very low, typically <5%, depending on its physicochemical properties [36]. Such low bioavailability is attributed to extensive precorneal drug loss by nasolachrymal drainage. The rapid elimination of the instilled drug often results in a short duration of therapeutic effect and, consequently, the need for a frequent dosing regimen [37]. Moreover, 50–100% of instilled dose may be absorbed systemically through drainage via the nasolachrymal duct. This could cause possible increased risk for undesirable side effects [36, 38]. For example, topical administration of beta-blockers for treatment of wide-angle glaucoma causes systemic side effects on the heart [39, 40]. Accordingly, systemic absorption of E_2_ solution drained through the nasolachrymal duct may increase the risk for undesirable side effects (e.g., increased risk of breast cancer, endometrial cancer, gynecomastia, etc.).

Delivery systems based on *in situ* gel-formation offer an attractive alternative to instillation of solutions because the in-situ gel increases pre-corneal drug residence time. The gelling process involves a phase transition in which the instilled solution forms a gel in the cul-de-sac of the eye as a result of response to some stimuli by the polymer. Therefore, these systems offer the dual advantage of an easy to administer liquid formulation along with the increased residence time of a gel. Parameters that can change and trigger this sol-gel phase transition include pH, temperature or ionic strength of the tear fluid. Among all *in situ* gel-forming systems, activation by change in ionic strength is most effective. The advantage is based on the fact that fluctuations in pH and temperature, which could cause changes in the gelation process, are not associated with the ion-activated system. These fluctuations in pH could cause ocular irritation, and storage conditions could lead to changes in temperature.

Numerous types of stimuli-responsive polymers used in ocular drug delivery systems have been reviewed previously [41]. Of all these systems, ion-activated gellan gum-based *in situ* gel systems have been shown to significantly prolong the ocular contact time of the drug in animal and human studies [42, 43]. Also, gellan gum (Gelrite®) based systems have been evaluated for many drugs, such as timolol maleate [44, 45], indomethacin and ciprofloxacin hydrochloride [46], pefloxacin mesylate [47] and gatifloxacin [48], pilocarpine hydrochloride [49], and Ketorolac tromethamine [50].

Gellan is an exocellular microbial heteropolysaccharide that is secreted by the strain *Pseudomonas elodea*. It is an anionic polymer with a high molecular weight (approx. 5×10^5^ daltons, deacetylated). The polymer is stable to both heat and pH (pH 3.5–10.0) [51]. The gellan gum being an anionic polymer forms clear gels in the presence of mono (Na^+^, K^+^) and divalent (Ca^2+^) cations of the tear fluids with good *in situ* gelling characteristics at as low as 0.1%(w/v) polymer concentration [52].

A key challenge in the development of a sterile ophthalmic *in situ* gel-forming solution eye drops lies in the ability to ensure that the formulation has acceptable characteristics of sterility, clarity, tonicity, drug release, viscoelasticity, and stability. Also, with E_2_ being a biopharmaceutical classification system (BCS) class-II drug (i.e., low aqueous solubility [0.03mg/L at 25°C] and high permeability [logP of 3.94]), its delivery via the ocular route as an *in situ* gel-forming solution is limited by its poor aqueous solubility. Therefore, the purpose of this study is to develop safe, sterile and stable *in situ* gel-forming estradiol solution eye drops for subsequent testing in humans and animal models to determine its role in cataract prevention and help elucidate the mechanisms by which estrogen protects lens transparency.

## Materials and methods

### Materials

Deacetylated gellan gum (Kelcogel^®^ CG-LA) was a free sample from CP Kelco (Atlanta, GA, USA). 17β-Estradiol (micronized), mannitol, potassium sorbate, and polysorbate 80 were of USP grade and were purchased from Spectrum Chemicals (New Brunswick, NJ). Reagent alcohol and acetonitrile were of HPLC grade and were also purchased from Spectrum Chemicals (New Brunswick, NJ). CoTran^TM^ 9711 microporous polyethylene (PET) membrane used for in vitro drug release studies was purchased from 3M (St Paul, MN). Water used for preparation of formulation was de-ionized and passed through a Milli-Q water purification system (Millipore, Bedford, MA, USA).

### Preparation of *in situ* gel-forming estradiol (E_2_) solution eye drops

The formulation consisted of 17β-estradiol (E_2_) (0.025% w/v), polysorbate-80 (4% w/v), Kelcogel^®^ CG-LA (0.3% w/v), mannitol (4.75% w/v) and potassium sorbate (0.3% w/v) and EDTA (0.03% w/v) [53]. Aliquot amounts of mannitol, potassium sorbate and edetate disodium dihydrate (EDTA) were accurately weighed and transferred to pre-weighed amount of Millipore water (~ 90 ml), while stirring in a glass beaker. After the solution was clear, the required amount of Kelcogel^®^ CG-LA was added and allowed to hydrate in the beaker. The preparation was then heated to 80°C and maintained until it turned clear. The clear solution obtained was then filtered through a Whatman^®^ filter No.1 (42.5mm Ø) and cooled to room temperature. After weight adjustment of the water loss due to evaporation, the preparation was sterilized by autoclave at 121°C and 15 psi pressure for 15 min. Estradiol stock solution containing aliquot of the drug in polysorbate 80 was prepared separately and added to the rest of the sterile preparation by filtering aseptically through 0.22μm sterilizing filter under a laminar flow hood. The resultant mixture was then stirred to obtain a homogenous, sterile, *in situ* gel-forming E_2_ ophthalmic solution.

### Formulation testing and stability evaluation

Pharmaceutical *in situ* gel-forming preparations intended for ocular administration should exhibit certain desirable characteristics to be suitable for human use and meet regulatory standards. These characteristics and criteria include clarity (visually clear), pH, (6–7) isotonicity (275–325 mOsm/kg) and antimicrobial efficacy for multi-unit dosage form (compliance with USP for Class-I products). The developed *in situ* gel-forming formulations were tested *in vitro* for these product performance characteristics. Also, the developed formulations were evaluated for drug stability at multiple time points and storage conditions (Room temperature (RT)-25°C ± 2°C/60% ± 5% RH; Intermediate (INT)-30°C ± 2°C/ 65% ± 5% RH; and Accelerated (ACC)-40°C ± 2°C/75% ± 5% RH) defined in ICH *Q1A (R2)* guideline on stability testing of new drug substances and drug products [54].

### *In vitro* characterization of *in situ* gel-forming E_2_ ophthalmic eye drops

#### Formulation pH

The pH of the formulations was tested using VWR-9100 pH meter. Prior to testing, the pH meter was calibrated using pH standards of 4, 7, and 10 ± 0.01 at 25°C. All formulations were tested for pH in triplicate and the mean value was calculated.

#### Osmolality testing

Osmolality of the formulations was tested using Wescor Vapor Pressure Osmometer Model-5520. Prior to use, the instrument was calibrated using standard solutions of 290, 1000, and, 100 mOsm/kg. Measurements were done in triplicate and mean value for each formulation was calculated.

#### Clarity measurement

Qualitative measure of the formulation’s clarity was first performed by visual observation against the light-dark background. Only those formulations that demonstrated clarity on visual observation were then quantified for their percent light transmission. A Perkin-Elmer UV-Vis spectrometer (Lambda 35 Model) was used to measure percent light transmission of the formulations in the visible region at 490 nm wavelength against water as a reference standard.

#### Drug (E_2_) potency

Drug (E_2_) content in the formulations was measured using a validated UV based stability-indicating gradient-HPLC method previously developed by our group. [55]. Reagent alcohol was used as a solvent for E_2_ and non-solvent for the polymer. For the extraction process, 50 μL of the formulation was diluted to 1 mL with the solvent in a 1.2 mL centrifuge tube. This mixture was then vortexed for a minute and sonicated for 15 min in a water bath maintained at 35°C. Further, the tubes were vortexed for another minute and centrifuged in a MicroV micro centrifuge for 5 min at 8000 rpm. The supernatant was then diluted and analyzed for E_2_ concentration. The HPLC system consisted of a Waters Alliance (Waters Corporation, Milford, MA, USA) equipped with a Waters 2690 separation module and Waters 2475 fluorescence detector. Data acquisition was performed by the Empower^TM^ 2 Pro software. Analysis was carried out at UV absorption wavelength of 280 nm with a Luna C_18_ (2) reversed-phase column of 250 mm × 4.6mm i.d., 5 μm dimensions (Phenomenex, Torrance, CA, USA) at ambient temperature. The mobile phase consisted of a gradient of acetonitrile and water 35:65 (v/v) ratios from 5.5 min to acetonitrile and water 50:50 (v/v) ratios in 18 min set at a flow rate of 1mL/min.

Drug stability and degradation upon storage at different temperatures and time points was characterized by treating the data to zero and first order reaction kinetic models. The integral forms of the zero-order and first-order reaction rates are shown in Eq 1 and Eq 2.

$$C_{t}=C_{0}-K_{0}\times t$$ Eq. 1

$$\ln\;C_{t}=\ln\;C_{0}-K\times t$$ Eq. 2

Where C_o_ and C_t_ are the concentrations at initial time and at time t. K_o_ and K are the zero-order and first-order reaction rate constants in units of concentration/time and (1/time), respectively. Accordingly, based on the order of the reaction, the shelf life defined as the time taken to reach 90% (t_90%_) of the initial drug concentration or time taken for 10% drug degradation was given by:

$$\text{For zero-order reaction},\;t_{90\%}=\frac{0.1\times C_{0}}{K_{0}}$$ Eq. 3

$$\text{For first-order reaction},\;t_{90\%}=\frac{0.105}{K}$$ Eq. 4

#### Antimicrobial efficacy

Formulations were tested according to USP 31 <51> Antimicrobial effectiveness testing procedure for USP Class-I products (i.e., ophthalmic and otic preparations). In this test, aliquots of each formulation were inoculated with 10^5^−10^6^ cfu/mL concentration of *Pseudomonas aeruginosa*, *Escherichia coli*, and, *Staphylococcus aureus* and incubated for 28 days at 22.5 ± 2.5°C. The efficacy of preservatives in inhibiting the growth of microorganisms was tested during storage at 7, 14, and 28 days. According to USP 31 <51>, the preservative in the formulations are considered to be antimicrobially effective only if there was a “*1log*” decrease in the concentration of the microorganisms at day 7, “*3log*” decrease in the concentration of the microorganisms at day 14 and no significant change in the concentration of microorganisms from day 14 to day 28.

#### Rheological characterization

Flow Behavior: Rheological flow characterization of the developed formulations was conducted with AR 2000 rheometer (TA Instruments, New Castle, Delaware, USA) using double-concentric cylinder geometry and steady state peak-flow method. The instrument was operated according to the standard operating procedure. 6.5 mL of the formulation was transferred to the geometry and apparent viscosity (η_app_) at 25°C was determined using peak hold step, in which a constant shear stress was applied and the corresponding shear rate was recorded for 45 seconds period. This peak-hold step was further repeated at various constant shear stress and the corresponding apparent viscosities and shear rates were recorded. The ranges of shear stress investigated were those that would result in shear rates range of 10–1000 s^-1^ that a formulation typically experiences in the eye at rest and during blinking [56]. The flow behavior of the formulation was then characterized by fitting the data obtained to the Ostwald Power-law rheology model.

$$\mu_{app}=\frac{\tau }{\dot{\gamma }}=K{\dot{\gamma }}^{n-1}$$ Eq. 5

Where *μ*_*app*_ is apparent viscosity, *τ* is shear stress, *γ* is shear rate, *K* is consistency coefficient, and *n* is flow index.

Viscoelasticity: Viscoelastic characterization of the developed formulations was conducted with AR 2000 rheometer (TA Instruments, New Castle, Delaware, USA) using double-concentric cylinder geometry and dynamic small amplitude oscillatory rheometry (SAOR). To simulate the *in vivo* interaction of the administered *in situ* gel-forming eye drops with the conjunctival tear fluid, the formulation was pre-mixed with simulated tear fluid (STF) in 30:7 (v/v) ratios. About 6.5 mL of the resulting mixture was transferred to the double-concentric cylinder geometry and analyzed for viscoelastic parameters. The STF was composed of 6.8 g sodium chloride (NaCl), 2.2 g sodium bicarbonate (NaHCO_3_), 0.084 g calcium chloride dehydrate (CaCL_2_.2H_2_O), 1.4 g potassium chloride (KCL) in 1 L of ultra pure water. These amounts results in the mono and divalent ion concentrations of 142 mM of Na^+^, 19 mM of K^+^, and 0.6 mM of Ca^2+^ that are comparable with ionic contents of the tears [52]. The pH of the STF was then adjusted to 7.4 using 1N HCl. Prior to testing; the double-concentric cylinder geometry was preheated to 35°C, to make measurements at the physiological eye temperature.

In this study, the oscillatory stress sweep measurements are carried out first to determine the linear viscoelastic region (LVR) of the sample and, therefore, the consequent choice of the stress or strain value to use in the oscillatory frequency sweep test. *In situ* gel structure formation and viscoelastic behavior were then confirmed by analyzing the frequency dependency of the viscoelastic indices obtained from the frequency sweep test by fitting to the power-law rheology model shown below.

$$G^{\prime }(\omega )\;\alpha \;\omega^{n}$$ Eq. 6

Where, G’ is elastic modulus, ω is angular frequency, and *n* is power law coefficient [57].

#### *In vitro* drug release studies

Drug release from the developed *in situ* gel-forming E_2_ formulations was studied using modified USP-XXXII Type-II dissolution apparatus and Enhancer Cells™. The test was performed at 100 rpm paddle speed and 35°C temperature. Simulated tear fluid (200 mL, pH 7.4, and 30% v/v ethanol) maintained at 35 ± 0.2°C was used as dissolution medium. One mL of the test formulation was transferred to the Enhancer Cell™ reservoir using a micropipette followed by covering of the transferred material with a thin CoTran™9711 polyethylene membrane (3M, St Paul, MN) previously soaked overnight in the dissolution medium. The membrane was then secured in place using sealing ring and patch retainer. The Enhancer Cell™ was then carefully transferred to the dissolution vessel and drug release was measured at different time points of 0.5, 1, 2, 3, 4, 6, 8, 10 and 24 hr. At each time point, a fixed volume (i.e., 2 mL) of the dissolution medium was sampled and replaced by the fresh dissolution medium. The drug (E_2_) concentration in the dissolution media at each time point was measured using a validated HPLC assay method discussed earlier. Using the drug (E_2_) release data obtained at different time points, the release kinetics were characterized by treating the data to drug release kinetic models.

#### Release kinetics

To elucidate the mechanism of E_2_ release from the *in situ* forming hydrogels, the drug release data was characterized using the power-law model proposed by Korsemeyer-Peppas [58] (shown in Eq 7) and the time for 20% (t_20_) and 80% (t_80_) of drug release were compared for statistical significance using ANOVA.

$$\frac{M_{t}}{M_{\infty }}=Kt^{n}$$ Eq. 7

Where, *M*_*t*_*/M*_*∞*_ is the fraction of drug released in time t, *K* is drug release rate constant, which incorporates structural and geometrical characteristics of the controlled release system, and *n* represents the release exponent indicative of mechanism of drug release. When n = 0.5, the drug diffuses through and is released by a quasi-Fickian diffusion mechanism. For 0.5>n<1.0, non-Fickian solute diffusion is observed and when n = 1, the diffusion is swelling controlled and is termed pseudo-Case II solute transport.

Also, the differences in the drug release profiles at different storage conditions and times during storage were analyzed by computing similarity factor, f_2_ proposed by Moore and Flanner [59]. The similarity factor is a statistic that measures the closeness of between two dissolution profiles and is given by Eq 8.

$$f_{2}=50\times \log\;\left\{{\left[1+(\frac{1}{n})\sum_{t=1}^{n}{\left(R_{t}-T_{t}\right)}^{2}\right]}^{-0.5}\times 100\right\}$$ Eq. 8

Where *n* is the number of time points, *R*_*t*_ is the dissolution value of the reference product at time t, and *T*_*t*_ is the dissolution value of the test product. The larger the value of f_2_ or the closer the value of f_2_ is to 100, the smaller is the difference between the two curves. The FDA suggests that the two dissolution profiles are considered to be similar if the f_2_ similarity factor is between 50 and 100 [60]. The lower acceptable value (i.e., 50) corresponds to 10% average absolute difference between a reference product and a test product at each time point.

### Data analysis and interpretation

The parameter estimates obtained for different product performance characteristics at different time points and storage conditions were compared and analyzed for statistical significance by ANOVA. The significance level (α) for all analyses was ≤0.05. All statistical analyses were conducted in GraphPad^®^ Prism (Version 5, GraphPad, San Diego, CA).

## Results and discussion

Based on pre-formulation studies, levels of polysorbate-80 (4% w/v) and mannitol (4.75% w/v) required to achieve target E_2_ concentration and osmolality values, respectively, were identified. Gellan gum being a polysaccharide can sustain microbial growth. Hence, preservatives were necessary to provide sterility and prevent bacterial growth during formulation storage and administration. Potassium sorbate and EDTA were chosen as preservatives in the formulation as they were found to be compatible with other excipients in the formulation. Potassium sorbate was chosen as a suitable preservative based on the literature evidence that it was poorly taken up by the micelles of polysorbate-80, the surfactant. Potassium sorbate also provided satisfactory preservative effect of ophthalmic solutions [61]. Addition of EDTA to the formulations containing potassium sorbate had been shown to increase preservative effect [61–63]. Presumably, EDTA binds to the essential minerals in the microbial cytoskeleton and disorganizes its assembly, thereby, enhancing the permeability of potassium sorbate resulting in a synergistic antimicrobial effect. Accordingly, potassium sorbate and EDTA were chosen as preservatives in the formulation at concentrations of 0.3% w/v & 0.03% w/v, respectively.

The critical product attributes of the developed *in situ* gel-forming E_2_ solution eye drops s are summarized in Table 1. The pH of the formulation at different storage conditions and time points were in the range of 6.35–6.36 units (see Table 1). The physiological pH of the tears is approximately 7.4. Although eyes can tolerate a fairly wide pH range (i.e., 4.5–8.5), the closer the pH of the formulation is to the physiological pH, the better it is tolerated. The pH values were stable during storage and storage conditions had no significant influence on the corresponding pH of the preparation at different time points (p>0.05, one-way ANOVA). The developed formulations remained clear throughout the six months stability storage period. The percent light transmittance of the eye drops in the visible range, at 490 nm wavelength, was >95% at all storage conditions and time points (see Table 1). The formulations were found to be isotonic. The osmolality of the formulation during storage was within the acceptable range of 275–325 mOsm/Kg (see Table 1). Analysis of the osmolality data obtained at different time points during storage at different conditions using one-way ANOVA indicated no significant differences (p>0.05). Also, the antimicrobial effectiveness test against Pseudomonas *aeruginosa*, *Escherichia coli*, and, *Staphylococcus aureus* revealed that the formulations were antimicrobially effective throughout the stability study duration. The sterility of the formulation was maintained during storage up to 6 months at all the storage conditions studied. The antimicrobial efficacy results demonstrated that the preservative combination of potassium sorbate and EDTA and their concentrations in the formulation were acceptable.

**Table 1:** Summary of critical characteristics of the optimized *in situ* gel-forming E_2_ eye drops at different storage conditions.

**pH (units)**				
Storage Condition	0	2 month	4month	6month
Long Term (RT)	6.36±0.01	6.36±0.02	6.35±0.01	6.36±0.02
Intermediate (INT)	6.36±0.01	6.36±0.02	6.35±0.03	6.36±0.03
Accelerated (ACC)	6.36±0.01	6.36±0.02	6.35±0.03	6.35±0.04
**Osmolality (mOsm/Kg)**				
Storage Condition	0	2 month	4month	6month
Long Term (RT)	289±3	291±2	289±2	290±4
Intermediate (INT)	289±3	289±4	287±3	288±4
Accelerated (ACC)	289±3	288±4	292±5	291±5
**Clarity (%UV transmittance)**				
Storage Condition	0	2 month	4month	6month
Long Term (RT)	97±2	96±2	96±3	95±2
Intermediate (INT)	96±1	95±2	96±2	95±2
Accelerated (ACC)	96±2	95±3	97±2	95±2
**Antimicrobial efficacy**				
Storage Condition	0	3month	6month
Long Term (RT)	Yes	Yes	Yes
Intermediate (INT)		Yes		Yes		Yes	
Accelerated (ACC)	Yes	Yes	Yes

### Apparent viscosity

**Fig 1:**
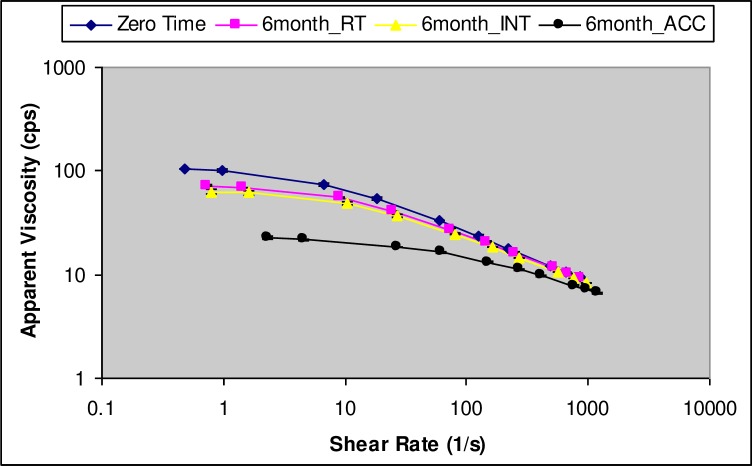
Flow curves showing the effect of storage conditions on apparent viscosity of the *in situ* gel-forming estradiol formulation.

The apparent viscosity of the developed *in situ* gel-forming formulation measured at the shear rate of 100s^-1^ decreased with increase in shear rate (see Fig 1). The formulation is considered shear thinning if its apparent viscosity decreased with increase in the applied shear rate. Such shear-thinning behavior was observed due to the presence of gellan gum polymer network in the formulation that provided low-resistance to flow upon shear. Also, shear thinning nature will allow for easy processing, handling, and administration of the *in situ* gel-forming ophthalmic solutions [56, 64]. The apparent viscosity of the formulation varied in the range of 12.5–23.2 cps depending upon the storage condition (see Table 2). The viscosity of the formulation decreased with increase in the storage temperature. However, the apparent viscosities were within the acceptance criteria (i.e., <50cps at 100s^-1^) throughout the study duration.

**Table 2:** Apparent viscosity of the optimized *in situ* gel-forming E_2_ eye drops at different time points during storage at different conditions.

Apparent Viscosity (cps) at 100s^-1^				
Storage Condition	0	2 month	4 month	6 month
Long Term (RT)	23.2±1.2	22.7±0.5	21.6±0.9	21.0±1.1
Intermediate (INT)	23.2±1.2	21.9±0.3	*20.4±0.2	*19.6±0.3
Accelerated (ACC)	23.2±1.2	20.9±0.3	*15.7±0.3	*12.5±0.6

*p<0.05; n = 3 in all cases.

Statistical one-way ANOVA analysis of the viscosity data using Prism^®^ indicated that long-term storage conditions did not have statistically significant (p>0.05) influence on the apparent viscosity of the formulation. Whereas, intermediate and accelerated storage conditions showed statistically significant influence on the apparent viscosity of the formulation at 4 and 6 month (p<0.05) time points. But, the apparent viscosities were still within the acceptance criteria, and hence, formulations are considered stable.

### Viscoelasticity

**Fig 2:**
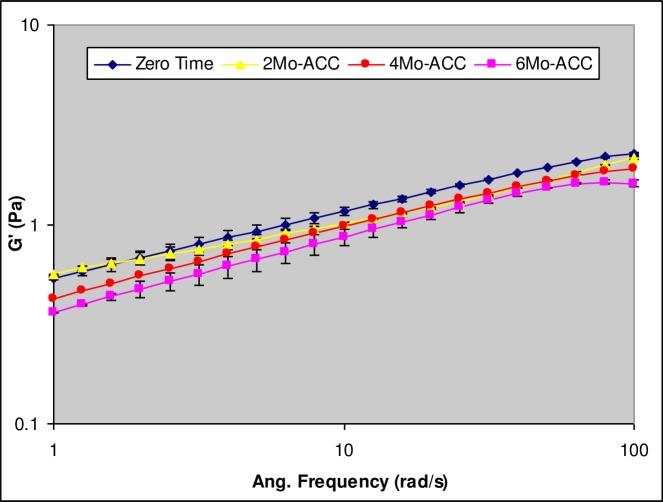
A representative frequency sweep plot showing the effect of accelerated (ACC) storage on the elastic modulus (G’) of the *in situ* gel-forming E_2_ solution eye drops.

Oscillatory measurements were carried out to identify linear viscoelastic region (LVR) for the characterization of developed *in situ* gel-forming solution. For this study, the conditions for LVR were identified as 3% strain and 5 rad/s angular frequency. A representative frequency sweep plot of the developed *in situ* gel-forming E_2_ solution eye drops measured in the presence of STF (30:7 v/v ratios) at different time points during storage at the accelerated conditions is shown in Fig 2.

The power-law rheology model fitting parameters and the viscoelastic parameter estimates of the developed formulation obtained during storage are similarly summarized in Table 3. The developed formulation showed evidence of phase transition and *in situ* gel-structure formation in the presence of STF at all the time points during storage as G’>G”, δ<45° (see Table 3). The mechanism of gelation involved reduction in electrostatic repulsions between the carboxyl groups of the gellan gum chains in the presence of cations of the STF; which promoted a coil-to-helix transition followed by helix aggregation to form a gel network [52, 65, 66]. Further, the frequency sweep plots (Fig 2) showed moderate to weak dependency of elastic modulus (G’) on angular frequency according to power-law rheology model with the model exponent “*n*” values in the range of 0.3–0.4 (Table 3). The *n* value in this range further confirms the nature of the material formed to be viscoelastic and that it corresponds to a gel state according to the acceptance criteria.

**Table 3:** Summary of viscoelastic parameter estimates and power-law rheology model fitting parameters of the *in situ* gel-forming E_2_ eye drops at different stability storage conditions.

Storage Condition	Time (month)	Viscoelastic indices in LVR of 5 rad/s and 3% strain			Model-fitting Parameters	
		G’ (Pa)	G” (Pa)	δ (°)	N	r^2^
**---**	0	0.92±0.01	0.49±0.01	27.7±0.5	0.3	0.99
RT	2	0.92±0.07	0.50±0.01	28.5±2.1	0.3	0.99
ACC	2	0.84±0.01	0.46±0.02	28.7±1.3	0.3	0.99
RT	4	0.85±0.02	0.40±0.01	25.2±0.2	0.4	0.96
ACC	4	*0.77±0.01	0.40±0.01	27.2±0.1	0.3	0.99
RT	6	0.84±0.01	0.46±0.02	28.7±1.3	0.3	0.99
ACC	6	*0.66±0.01	0.33±0.01	26.6±0.2	0.3	0.99

*p<0.05 (1-wayANOVA); n = 3 in all cases.

The magnitude of viscoelastic parameter estimates (i.e., G’ and G”) decreased with change in storage condition as well as length of storage (Table 3). The modulus of elasticity (G’) values, reflective of the strength of the gel structure formed, varied in the range of 0.85–0.92 Pa and 0.92–0.66 Pa during storage for 6 months at long-term (RT) and accelerated (ACC) conditions, respectively. Statistical one-way ANOVA analysis of the elastic modulus data indicated that long-term storage conditions of 25°C/60%RH did not have statistically significant (p>0.05) influence on the gel strength or viscoelasticity of the formulation throughout the study duration. In contrast, storage at accelerated conditions of 40°C/75%RH, resulted in statistically significant decrease in the gel strength or elastic modulus of the formulation at 4 and 6 months (p<0.05). Such decrease in gel strength of the formulation upon accelerated storage indicate the susceptibility of the polymer in the formulation to physically degrade and lose capacity to form stronger gel structures in the presence of simulated tear fluid at higher temperature and relative humidity (i.e., > 40°C/75%RH).

### Stability of estradiol (E_2_)

The results of E_2_ assay for the *in situ* gel-forming solution eye drops are summarized in Table 4. The E_2_ assay values at day 0 and day 180 varied in the range of 100.3%-99.3%, 100.3%-97.7%, and 100.3%-96.6% at RT, INT, and ACC storage conditions, respectively. At any storage condition, the degradation was <5% and no degradation products were detected using the stability indicating HPLC assay method, suggesting the stability of the optimized formulation. Statistical one-way ANOVA analysis of the assay data obtained for the developed formulation at different storage conditions indicated that only at 6 months storage at accelerated storage condition of 40°C/75%RH, the E_2_ assay was statistically significant (p<0.05) compared to the initial assay value. Although significant, the E_2_ assay values of the formulation were within the specification limit of 90–110% throughout the study duration at all tested storage conditions, and hence considered highly stable and desirable.

**Table 4:** Stability of E_2_ from the developed *in situ* gel-forming solution eye drops stored at different stability storage conditions.

Storage Time	E_2_ Concentration (Mean % ± SD)		
	Storage Condition		
	RT	INT	ACC
Zero/Initial	100.3 ± 1.2	N/A	N/A
1-Month	99.4 ± 0.7	99.9 ± 1.1	100.0 ± 3.3
2-Month	99.1 ± 0.9	99.1 ± 2.4	97.8 ± 2.6
3-Month	99.0 ± 1.9	98.9 ± 1.6	98.4 ± 1.2
4-Month	99.7 ± 3.2	98.3 ± 1.9	97.9 ± 1.4
6-Month	99.3 ± 1.5	97.7 ± 1.5	*96.6 ± 1.2

*p<0.05(1-wayANOVA).

**Fig 3:**
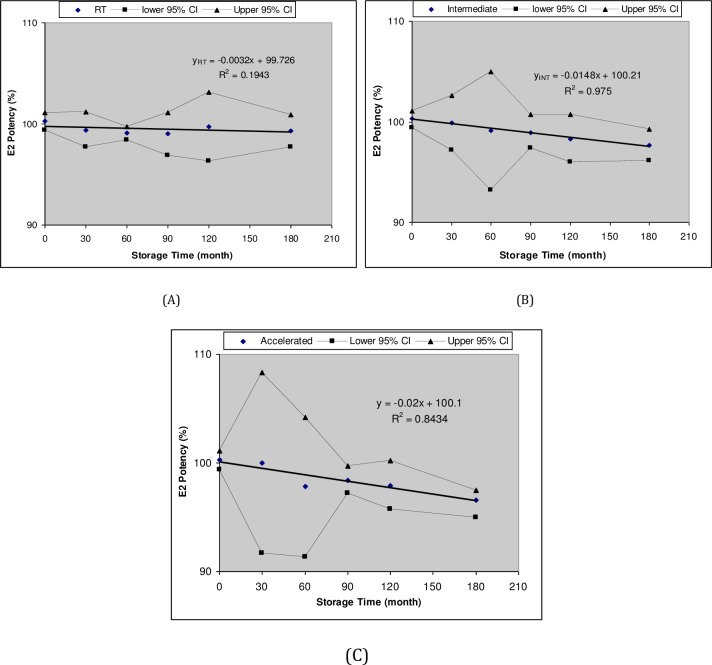
Zero-order degradation kinetics of E_2_ *in situ* gel-forming solution eye drops stored at A) Room temperature B) Intermediate C) Accelerated storage conditions.

The E_2_ degradation in the solution eye drops followed zero-order kinetics at all tested storage conditions (Fig 3). The degradation rate constant at real-time (RT) storage condition was 0.003%/day and the estimated shelf-life (t_90%_) was 3323 days or 9.1 years (see Table 5). The degradation rate constant at intermediate (INT) storage condition was 0.015%/day and the estimated shelf-life (t_90_) was 668 days or 1.8 years (see Table 5). The degradation was relatively faster at accelerated (ACC) storage condition with rate constant of 0.02%/day and estimated shelf-life (t_90_) of 500 days or 1.4 years (see Table 5). Such long shelf-life at ACC storage conditions indicates acceptable stability of the formulation. ICH guidelines recommend a tentative shelf-life of 2 years at room temperature if the product remains stable and drug assay varied within the acceptance limits of 90–110% at accelerated storage condition for 6 months [54, 67]. Accordingly, a tentative shelf-life of 2 years can be assigned for the developed *in situ* gel-forming E_2_ solution eye drops for storage at room temperature.

**Table 5:** Summary of zero-order degradation rate constants for the developed E_2_ *in situ* gel-forming solution eye drops stored at different conditions for 6 months.

Storage Conditions					
RT		INT		ACC	
*K*_*o*_(%/day)	t_90_(days)	*K*_*o*_(%/day)	t_90_(days)	*K*_*o*_(%/day)	t_90_(days)
0.003	3323	0.015	668	0.020	500

### Drug release characterization

**Fig 4:**
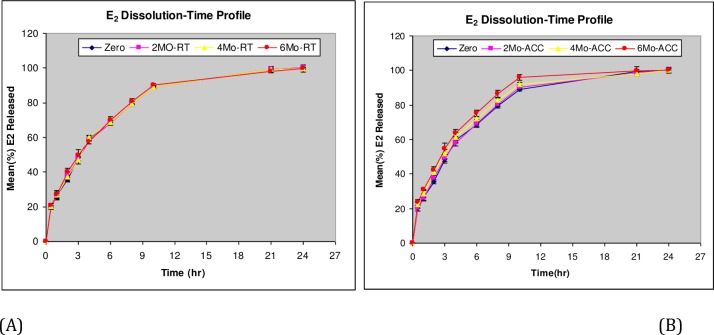
Cumulative drug release profiles of E_2_ from the *in situ* gel-forming solution eye drops stored at A) Room Temperature; B) Accelerated storage conditions.

The cumulative E_2_ release profiles from the developed *in situ* gel-forming solution eye drops at different time points during storage at real-time (RT) and accelerated (ACC) conditions are shown in Fig 4. The drug release was found to be sustained with 80% of the drug released in about 7–8 hr. An initial faster release of the drug from *in situ* gel-forming solutions was also observed with 20% of the drug released in 0.4–0.5 hr.

**Fig 5:**
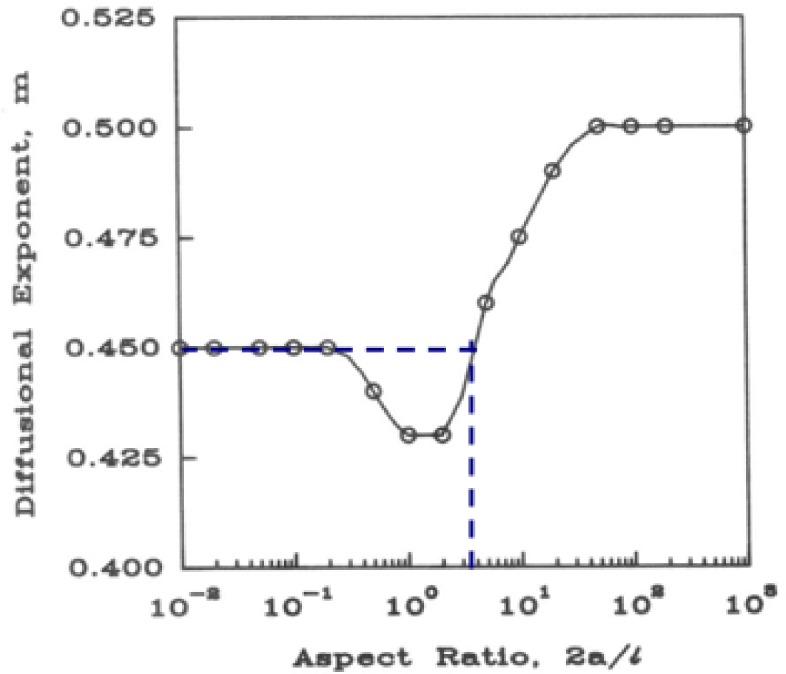
Variation of the Fickian diffusional exponent, *m*, with the aspect ratio, *2a/l*, where *2a* is the diameter and *l* is the thickness (height) of the device (Peppas et al., 1989)

The model fitting parameters and drug release estimates (i.e., t_20%_ and t_80%_) obtained upon treating the drug release data to Korsemeyer-Peppas Model are summarized in Table 6. The release rate constant (*K*) was in the range of 27.4–28.4(%h^-n^) at real-time (RT); whereas for the accelerated storage (ACC) condition, it was in the range of 27.4–31.8 (%h^-n^). The magnitude of the release exponent *n* was in the range of 0.50–0.51 at real-time storage condition; whereas, for the accelerated storage (ACC) condition, it was in the range of 0.48–0.52. The cut-off value of *n* for a purely Fickian diffusion mechanism in case of gel systems with aspect ratio of 3.6 (i.e., *2a* = 21.34mm and *l* = 5.96mm) was 0.45 (Fig 5). The *n* values >0.45 suggest that **a)** E_2_ release from the gels during storage was function of drug diffusion from the polymer gel matrix as well as polymer relaxation, and **b)** E_2_ release kinetics or mechanism of drug release was not significantly influenced by the storage conditions.

**Table 6:** Summary of model fitting parameters and drug release estimates of E_2_ from the *in situ* gel-forming solution eye drops.

Storage Time	Storage Condition	*K*	*n*	r^2^	t_20_ (hr)	t_80_ (hr)
Zero	25°C/60%RH	27.4±0.3	0.51±0.01	0.98	0.54±0.02	8.12±0.04
2-month	25°C/60%RH	27.4±0.6	0.51±0.01	0.99	0.54±0.03	8.15±0.05
	40°C/75%RH	27.4±0.1	0.52±0.01	0.99	0.54±0.01	7.93±0.06
4-month	25°C/60%RH	27.7±0.2	0.51±0.01	0.98	0.53±0.01	8.08±0.02
	40°C/75%RH	30.1±0.2	0.49±0.01	0.99	0.43±0.01	7.49±0.11
6-month	25°C/60%RH	28.4±0.1	0.50±0.01	0.99	0.51±0.02	7.85±0.04
	40°C/75%RH	31.8±0.3	0.48±0.01	0.99	0.38±0.01	6.84±0.06

As shown in Table 6, the time for 20% or 80% of the drug to be released (t_20%_) or (t_80%_), respectively, decreased with storage condition and storage time. At room temperature, t_80%_ decreased from 8.1 hr at day 0 to 7.9 hr at 6 months; whereas, for the accelerated storage condition, t_80%_ decreased from 8.1 hr at day 0 to 6.8 hr at 6 months. Faster drug release at accelerated condition was due to concomitant decrease in the strength of the gel-structure (i.e., G’) at higher temperature (i.e., 0.94 Pa at day 0 vs. 0.66 Pa at day 180 at accelerated condition).

The similarity factor (f_2_) was used to compare E_2_ dissolution profiles from the developed *in situ* gel-forming solution at day 0 and at different time points during storage at 25°C/60% RH (RT) as well as 40°C/75%RH (ACC). The f_2_ results are summarized in Table 7. The f_2_ values for E_2_ drug release upon storage at RT at 2, 4, and 6 months was 93, 94, and 82, respectively. Similarly, the f_2_ values for E_2_ drug release upon storage at accelerated condition at 2, 4, and 6 months was 91, 74, and 62, respectively. The f_2_ values in the range of 50–100 at each time point and storage condition suggest that the drug release profiles are statistically similar (60 SUPAC-IR, 1995). However, the larger the value of f_2_ or the closer the value of f_2_ is to 100, the smaller is the difference between the two curves. Hence, it can be concluded that the differences in the drug release profiles upon storage at 25°C/60% RH (RT) were smaller compared to storage at 40°C/75%RH (ACC).

**Table 7:** Comparison of E_2_ dissolution profiles of the *in situ* gel-forming eye drops during storage using similarity factor (f_2_).

Storage Time	Similarity Factor (f_2_)	
	25°C/60%RH (RT)	40°C/75%RH (ACC)
2-month	93	91
4-month	94	74
6-month	82	62

The formulation was evaluated for ocular irritation and pharmacokinetic profile in rabbits (manuscript in preparation). The absorption of E_2_ into the eyes, as shown by the area under the curve (AUC) in aqueous humor, was 250-fold higher than in the systemic circulation (serum). This enhanced ocular absorption was due to the the viscoelastic nature of the gel that caused prolonged pre-corneal drug residence time and consequential reduction in drug drainage through nasolacrimal duct. There was no clinically significant irritation or toxicity in the eyes or other ocular tissues in rabbits. Additionally, to examine E_2_ delivery and actions within the lens itself, this ocular formulation was tested in ERΔ3 transgenic mice that develop cataracts after exposure to exogenous estrogens due to expression of an estrogen receptor repressor [21]. Ocular administration of the *in situ* gel-forming formulation confirmed localized delivery and activity of E_2_ by inducing cataracts and lenticular estrogen-responsive genes in ERΔ3 mice (manuscript in preparation).

## Conclusions

An *in situ* gel-forming E_2_ solution eye drop was successfully developed using gellan gum as an ion-activated polymer. The developed formulation was stable at room temperature and accelerated storage conditions for six months and exhibited acceptable pH, clarity, osmolality, sterility and antimicrobial efficacy. The formulations showed evidence of phase transition and *in situ* gel structure formation upon contact with cations of the simulated tear fluid. The *in situ* gel-formed was viscoelastic in nature and sustained the drug release for 7–8 hours. The drug release from the *in situ* gel-formed was governed by its diffusion from the gel matrix as well as polymer erosion. The comparison of E_2_ release data using similarity factor (f_2_) indicated that the drug release profiles from the developed formulation were similar throughout the stability study. The storage conditions did not show significant influence on the drug release and viscoelastic parameter estimates. As the drug degradation was <5% throughout the stability study, a tentative shelf-life period of 2 years was assigned to the developed drug product. In summary, the developed formulation was found to be stable with viscoelatic and drug release properties that could provide prolonged release and increased contact time. To be published in vivo data demonstrates that the formulation is safe and effective in preclinical models.
